# Application of livelihood vulnerability index to assess risks for farmers in the Sukoharjo Regency and Klaten Regency, Indonesia

**DOI:** 10.4102/jamba.v11i1.739

**Published:** 2019-07-15

**Authors:** Suryanto Suryanto, Aulia Rahman

**Affiliations:** 1 Department of Economics Development, Faculty of Economics and Business, Sebelas Maret University, Surakarta, Indonesia

**Keywords:** poverty, food security, livelihood vulnerability index, flood, climate change

## Abstract

This research tried to compare the level of farmers’ livelihood vulnerability to flooding in Sukoharjo and Klaten. Farmers are the most susceptible caused by climate change. The data used in this research are primary data, collected by interviewing 61 respondents who are farmers in the Sonorejo Village, Sukoharjo Regency and 72 respondents in the Jiwo Wetan Village, Klaten Regency. This data obtained by using non-probability sampling technique with purposive methods. Meanwhile mapping for hazard level was analysed by using Geographic Information System (GIS). Descriptive statistic was used for the livelihood vulnerability index’s (LVI) and the LVI-Intergovernmental Panel on Climate Change’s (IPCC) index calculation. The results show that the farmers’ livelihood vulnerability in the Sonorejo Village is medium level because of climate change based on the LVI index value at 0.363 and LVI-IPCC index value at 0.044. Meanwhile, the Jiwo Wetan Village has a lower index in LVI at 0.344 and LVI-IPCC index value at 0.038. Areas with similar physical characteristic and most of its community have a dependence on agriculture tend relatively the same level of vulnerability.

## Introduction

The increase in emissions of greenhouse gases (GHG) leads to an increase in temperature of the earth and causes global climate change. This is one of the key elements in the system of metabolism and physiology of plants; the global climate change has a major impact on the agricultural sector. According to the Agricultural Research and Development Agency (BBSDLP 2011), the agricultural sector is one of the most threatened sectors that is suffering and vulnerable to climate change. Salinger (2005) and Karim, Jahan and Islam (2014) stated three main factors in the agricultural sector based on global climate change, that is, (1) changes in the patterns of rainfall, (2) the rise of extreme climate events (floods and droughts) and (3) an increase in air temperature.

An extreme climate change causes a great impact on the agricultural sector. The climate change severely leads to changes in pattern, and the level of precipitation affects the initial time of planting and growing period. The alteration in level of precipitation affects the time and seasons, cropping patterns, land degradation, destruction of crops and productivity, planted and harvested acreage, and the damage to biodiversity, especially crops that is relatively sensitive to water availability (Runtunuwu & Syahbuddin 2007). Flooding on rice fields causes a reduction in harvest area and a decline in rice production significantly. High rainfall causes losses to farmers in the United States. The US EPA (2015) reported losses in 2010–2011, which reached $220 million. Mall, Gupta and Sungkar (2017) said that farmers must be given guidance to adapt because climate change has a negative effect.

Central Java Province is the second largest province of East Java with total area of wetland that is prone to flood or inundation (Table 1). Even so, Central Java Province remains one of Indonesia’s largest rice suppliers. Sukoharjo sub-district is one of the regions that largest suppliers of rice and food buffer in Central Java. Rice is the main agricultural product in Sukoharjo sub-district. According to report from the Minister of Agriculture and the Central Bureau of Statistics in Sukoharjo 2015, rice production in the Sukoharjo sub-district ranks third after the Polokarto sub-district and Mojolaban sub-district. Sukoharjo sub-district consists of 14 villages, where most of the land is used as a wetland. The use of paddy fields in the sub-district of Sukoharjo in 2013 amounted to 2.363 hectares (ha) or 53% of the total area of its territory. Sonorejo is the largest area with land use for rice fields; proportion is reached to 68% (302 ha) of the total area (444 ha). However, at some point the fields contained in the Sonorejo Village is prone to floods. However, Klaten is also a producer of rice in Central Java. Rice production in Klaten reaches 350 000 tons per year, while local demand is only 207 500 tons per year. Unfortunately, some areas become prone to flood because of overflow of the Dengkeng River. One of the worst impacted areas is the Jiwo Wetan Village in Klaten Regency, where, in 2017, there was a crop failure of 35.2 ha which was suffered by 123 farmers.

**TABLE 1:** Summary of research methods.

Research purposes	Analysis tools	Data	Results
Mapping of flood-prone wetland in the Sonorejo Village, Sukoharjo Sub-district	GIS	Primary data Secondary data	Map of flood vulnerability
Livelihood vulnerability index of farmers in the Sonorejo Village, Sukoharjo sub-district	Descriptive statistics	Primary data	Vulnerability index (livelihood vulnerability index)

GIS, Geographic Information System.

Based on the results of the mapping with Geographic Information System (GIS) and interview conducted with the Head of Village and the Head of Sonorejo’s Farmers Group Association (Gapoktan), wetland that is mostly prone to flood is located in the eastern part of the Sonorejo Village, namely, Langsur and Ngiser, that is directly adjacent to the Bulakrejo and Sukoharjo Village. The land altitude in those areas is lower than the surrounding areas, and the water drains (drainage) are not functioning properly, resulting to the flooding in the field.

The research area in Klaten Regency also has characteristics similar to the sample area in Sukoharjo Regency. The altitude of the Jiwo Wetan Region is lower than other areas around it. When the Dengkeng River overflows, the overflow leads in the terrain in the south of the village. If the inundation does not pass, then the water goes to the rice fields and settlements of the Jiwo Wetan Village.

The majority of the population in the Sonorejo and Jiwo Wetan villages rely on agriculture, especially crops and paddy rice. This condition causes the vulnerability on the livelihoods of farmers and their households. This study aims to deepen the understanding of the vulnerability of people’s livelihoods in flood-prone areas in paddy field in the Sonorejo Village and Jiwo Wetan Village, which are based on the results of the mapping with GIS and livelihood vulnerability index (LVI). The benefit of this research is to find out whether areas that have the same level of hazard and the same dependence on agriculture will have the same level of vulnerability.

## Methods and data analysis

Geographic Information System is used as a tool to find the area that is prone to flood. Thereafter, the analysis of the vulnerability of households’ farming based on flood occurrence will be conducted by using LVI calculations, which is based on the primary data obtained. Furthermore, the results are described as follows.

### Analysis by Geographic Information System

The utilisation of GIS has been done to identify areas of potential disasters. Wood and Good (2004) identify vulnerability at airports and seaports as catastrophic earthquake and tsunami. Saptutyningsih and Suryanto (2011), measure the environmental and social impact caused by natural disasters (ND). This research used the GIS to map the flood-prone areas in the Sonorejo Village and Jiwo Wetan Village by taking some variables into account, such as flood event data, curvature of the river, slope and land use.

### Livelihood vulnerability index approach

Vulnerability is one of the factors that determines whether people have risks to their livelihoods or not. According to IPPC (2007), the vulnerability assessment measures the ability of the community to respond to hazard and or secure their livelihood. Therefore, the index is used for comparison among the communities. Vulnerability Index or LVI was developed by Hahn, Riederer and Foster (2009), Madhuri, Thewari, and Bhowmick (2014), Simane, Zaitchik and Foltz (2016) and Richardson et al. (2018). This study wants to measure the vulnerability of households living in areas classified as flood-prone in Sukoharjo Regency and Klaten Regency. Most of the LVI studies were conducted to see differences in the level of vulnerability in different regional characteristics. Some examples of studies, Hahn et al. (2009) use LVI for coastal and inland populations, Simane et al. (2016) see differences in the characteristics of highland and lowland ecosystems and Richardson et al. (2018) further see LVI as a basis for predicting food security in a region. Nevertheless, the LVI calculations by Hahn et al. (2009) is applied in this study, which consists of the following seven main components:

- Socio-demographic profile (SDP)
- Livelihood strategies (LS)
- Health (H)
- Food (F)
- Water (W)
- Social networks (SN)
- Natural disasters (ND) and climate variability

According to Hahn et al. (2009), LVI component consists of several sub-components or indicators. These sub-components are developed based on a literature study of each major component. Table 2 shows how each of these sub-components is taken into account and limits the ability or potential bias.

**TABLE 2:** Design of the livelihood vulnerability index.

Major component	Sub-components	Explanation of sub-components	Potential limitations
Socio-demographic profile	Dependency ratio	The ratio of population < 15 and > 65 years of age to the population between 19 and 65 years of age	Large extended families, confusion about who is a member of the household, the absence of birth certificates
	Percentage of female-headed households	Household percentage of adult female. If the head of her family had no home > 6 months a year	Unable to determine the head of the family at home because there is more than one family living together without husband
	Percentage of households where head of household did not attend school	Percentage of heads of households who do not attended schools	-
	Percentage of households with members needing dependent care	Percentage of members of the house for at least a person who needs daily care, for instance, old age person, physical or mental condition and disability	-
Livelihood strategy	Percentage of households with family member working in a different city	Percentage of households whose members at least one person working outside the city for their primary work activity	Confusion about who the members of his family, did not count how many family members who have previously worked out of the city
	Percentage of households that the main income depends on natural resources or agriculture	Percentage of households that report the main income source only from agricultural sector	Only three main sources included
	Average Agricultural Livelihood Diversification Index (range: 0.20–1)	The opposite of (amount of agricultural activity +1), for example, household farming, gardening and farming, will have a livelihood classification index = 1/(3 + 1) = 0:25	Three main sources of family income in the agricultural sector. Outside the agricultural sector were not included
Health	Average time to health facility (min)	Average time required to reach the members of the house nearest health facility	Subjective estimation of travel time
	Households percentage with family member having chronic illness	Households percentage report with at least one family member having chronic illness	Subjectively defined by respondent
Food	Percentage of households dependent on family farm for food	Percentage of households that have food primarily from their personal farms	Subjectively defined by respondent
	Average number of months households struggle to find food (range: 0–12)	Average number of months households struggle to obtain food for their family	Subjective definition of ‘struggle’; believe in self-reported
	Percentage of households without crops savings	Percentage of households without crops savings from each harvest	Families that sell crops and save money are not counted
Water	Percentage of households that are using the source of natural water	Percentage of households that report their primary source of water is from river, lake or hole	Confusion regarding when families have more than one water source
	Average time to source of water (min)	Average time of households taken to travel to their primary source of water everyday	Subjective estimates of travel time
	Percentage of households without a consistent water supply	Percentage of households that report water is not available at their primary source everyday	Recall bias (more likely to remember several consecutive days of water shortage)
	The inverse of the average number of litres of water stored per household (range: > 0–1)	The inverse of (the average number of litres of water stored by each household +1)	Lack of information about the size of containers
Social networks	Average receive: Given ratio (range: 0–15)	The ratio of (how much help received by household in the past month +1) to (the number of types of help given by a household to someone else in the past month +1)	Confusion who is the family (immediate) and who is a relative (extended), reliance on self-reported types of help
	Average borrowed: Lend money ratio (range: 0.5–2.0)	The ratio of household borrowing money in the past month to a household lending money in the same month, for example, if a household borrowed money but did not lend money, the ratio = 2:1 or 2 and if they lent money but did not borrow any, the ratio = 1:2 or 0.5	Reliance on self-reported money exchanges, does not consider exchange of non-monetary goods
	Percentage of households that does not seek for assistance to their local government in the past 12 months	Percentage of households that reported they did not ask their local government for any assistance in the past 12 months	Confusion of believe in self-reported about the frequency of visits local government in past 12 months
Natural disaster and climate variability	Average number of floods, in the past 5 years (0–7)	Total number of floods incident that was reported by households in the past 5 years	Not remember about small flood event
	Percentage of households that did not receive warning about the pending natural disasters	Percentage of households that did not receive a warning about the most severe flood event in the past 5 years	Subjectively defined
	Percentage of households with an injury or death as a result of the most severe natural disaster in the past 5 years	Percentage of households that reported either an injury or death of any of their family member as a result of the severe flood in the past 5 years	Not remember small injures
	Standard deviation average mean of the daily maximum temperature by month	Standard deviation average of daily maximum temperature by month between 2009 and 2013 for its province	Short period of time
	Standard deviation average mean of the daily minimum temperature by month	Standard deviation average of the daily minimum temperature by month between 2009 and 2013 for its province	Short period of time

*Source*: Hahn, M.B., Riederer, A.M. & Foster, S.O., 2009, ‘The livelihood vulnerability index: A pragmatic approach to assessing risks from climate variability and change: A case study in Mozambique’, *Journal of Global Environmental Change* 19(1), 74–88. https://doi.org/10.1016/j.gloenvcha.2008.11.002/.

Each of these sub-components is calculated with different scales; therefore, an index is needed to calculate all the components as a whole. Accordingly, the composite index approach was used to convert the scale of each sub-component derived from *The Life Expectancy Index* (UNDP 2007), which is calculated as follows:

$$index_{Sd}=\frac{S_{d}-S_{\min}}{S_{\max}-S_{\min}}$$ [Eqn 1]

where, S_*d*_ is the value of the sub-components of the area *d*, and *S*_min_ and *S*_max_ indicate the minimum and maximum values of each sub-components that is determined by the data from the study area. Once standardised, the sub-components are averaged by using the following formula, and then calculate the value of its main components.

$$M_{d}=\frac{\sum_{i=1}^{n}index_{S_{d_{i}}}}{n}$$ [Eqn 2]

The value of *M*_*d*_ is equal to one of the main components in the area *d* (SDP, LS, H, F, W, SN and ND). The *d*_*i*_ index reflects the value of the sub-components that are indexed by *i*. Based on these equations, the LVI grades can be obtained by using the following equation:

$${\text{LVI}}_{d}=\frac{\sum_{i=1}^{7}w_{M_{i}}M_{di}}{\sum_{i=1}^{7}w_{M_{i}}}$$ [Eqn 3]

or

$${\text{LVI}}_{d}=\frac{w_{SDP}SDP_{d}+w_{LS}LS_{d}+w_{SN}SN_{d}+w_{H}H_{d}+w_{F}F_{d}+w_{W}W_{d}+w_{NDC}NDCV_{d}}{w_{SDP}+w_{LS}+w_{H}+w_{SN}+w_{F}+w_{W}+w_{NDC}}$$ [Eqn 4]

where, LVI_*d*_ represents the index value for the susceptibility in area, *d*, measured by seven major components. WM_*i*_ represents the number of sub-components that reflect to the main component, which is equally contributed to the overall LVI (Sullivan, Meigh & Fediw 2002). The scale of LVI grades ranged from 1 to 3, where:

- 0 to 0.2 = not vulnerable
- 12:21 to 0.4 = vulnerable
- 0:41 to 0.5 = very vulnerable.

### Livelihood Vulnerability Index approach-Intergovernmental Panel on Climate Change

Livelihood Vulnerability Index-Intergovernmental Panel on Climate Change (IPCC) index is an alternative method used when calculating LVI according to the IPCC definition of vulnerability. Table 3 shows the composition of seven key components of LVI-IPCC approach. The population exposure in this research is measured based on the number of floods that occurred in the last 5 years, while climate variability is measured by the average standard deviation of monthly maximum and minimum temperature and a monthly precipitation over a period of 5 years. Adaptive capacity is measured by the demographic profile of the area (headed by women), occupation of LS and SN (the percentage of households who helped neighbours). Then, sensitivity is measured by determining the present food status, water assurance and the health status of a region. The index showed in Table 3 is also used to calculate LVI-IPCC. The calculation of LVI-IPCC is different from the main components of LVI combined. Firstly, all components will be combined by category plan in Table 3 by using the following equation:

$${\text{CF}}_{d}=\frac{\sum_{i=1}^{n}w_{M_{i}}M_{di}}{\sum_{i=1}^{n}w_{M_{i}}}$$ [Eqn 5]

where, CF_*d*_ represents the contributing factors according to IPCC (exposure, sensitivity or adaptive capacity) for area *d* (Sonorejo Village); *M*_*di*_ represents a main component for area *d*, which is indexed by *I*; WM_*i*_ represents the quality of the main component; *n* represents the number of the main components of each contributing factor. The combination of these three contributing factors is calculated using the following equation:

$$\text{LVI}-{\text{IPCC}}_{d}=(e_{d}-a_{d})*s_{d}$$ [Eqn 6]

where LVI-IPPC_*d*_ represents the LVI index in area *d* expressed by using the framework of the vulnerability of the IPCC; *e* represents the score of area *d* (equal as the main component of ND and climate variability); *a* represents adaptive capacity score of area *d* (weighted by the average of the main component, i.e. LS, socio-demographic and SN) and *s* represents sensitive score of area *d* (weighted by the average of the major components, i.e. health, food and water). The scale of LVI-IPCC range between (−1) and (−0.4) is not vulnerable; (−0.41) and (0.3) is vulnerable or moderate and (0.31) and (1) is very vulnerable.

**TABLE 3:** Categories of major components contributing factors by the definition of vulnerability according to Intergovernmental Panel on Climate Change for the calculation of the Livelihood Vulnerability Index-Intergovernmental Panel on Climate Change.

Contributing factors to vulnerability	Major components
Exposure	Natural disaster and climate variability
Adaptive capacity	Socio-demographic profile
	Livelihood strategies
Sensitivity	Social networks
	Health
	Food
	Water

*Source*: Hahn, M.B., Riederer, A.M. & Foster, S.O., 2009, ‘The livelihood vulnerability index: A pragmatic approach to assessing risks from climate variability and change: A case study in Mozambique’, *Journal of Global Environmental Change* 19(1), 74–88. https://doi.org/10.1016/j.gloenvcha.2008.11.002.

### Ethical considerations

Respect for the dignity of research participants was prioritised. Anonymity of individuals and organisations participating in the research were ensured.

## Result

### Results prone to flood mapping with Geographical Information Systems

#### Flood

The high intensity of rainfall in the Sonorejo Village causes the rice fields in the area vulnerable to flooding. Based on the mapping of flood-prone rice fields in the Sonorejo Village, the areas most prone to flooding in lowland rice fields are Langsur and Ngiser. Meanwhile, the region that has the same characteristic with the Sonorejo Village is the Jiwo Wetan Village in Klaten Regency. The Sonorejo and Jiwo Wetan villages have an annual flood risk; the frequency of flooding is 4 to 6 times each year. In 2017, farmers in the Jiwo Wetan Village have accepted agricultural insurance claims because their agricultural land was damaged by floods (i.e. 75%).

#### The curvature of the river

The curvature of the river is the ratio between the valleys and the length of the river. The river in the eastern of Sonorejo Village is a meandering river that flows over gently sloping ground that begins to curve back and forth across the landscape. Meandering river has asymmetric channels. The deepest part of the channel is found outside of each bend. The water flows faster in these deeper fragments and grinds down material from the riverbank. The water flows more gently in the shallow areas near the inside of each bend. The slower flow of water cannot carry much residue and deposits its load on a series of point bars. Meandering rivers erode residue from the outer curve of each meander bend and deposit it on an inner curve further downstream.

The characteristics in the Jiwo Wetan Village area have similarity with the Sonorejo Village. Although the meandering is not like in Sonorejo, yet Jiwo Wetan is potentially flooding because the south of the village is the terrain of the Pegunungan Seribu, Yogyakarta. When rainfall is high, rainwater flows into the Dengkeng River and has the potential to increase the vulnerability of the area in Jiwo Wetan Klaten.

#### Slope

Slope in the Sonorejo Village, Sukoharjo sub-district, shows that most areas in the Sonorejo Village are flat with slope level ranging between 2% and 6%, which leads to the conclusion theoretically that the Sonorejo Village is highly susceptible to flooding. Slope in the Jiwo Wetan Village, Klaten Regency, is approximately 2% – 7%.

#### Land use

Land use index is determined by a map of land use and land use information on RBI topographic maps. The data collected are then contributed to the mapping of land use. The result shows that the Sonorejo and Jiwo Wetan villages are classified into three categories of land use, that is housing, irrigated fields and farming.

#### Potential flooding

**FIGURE 1:**
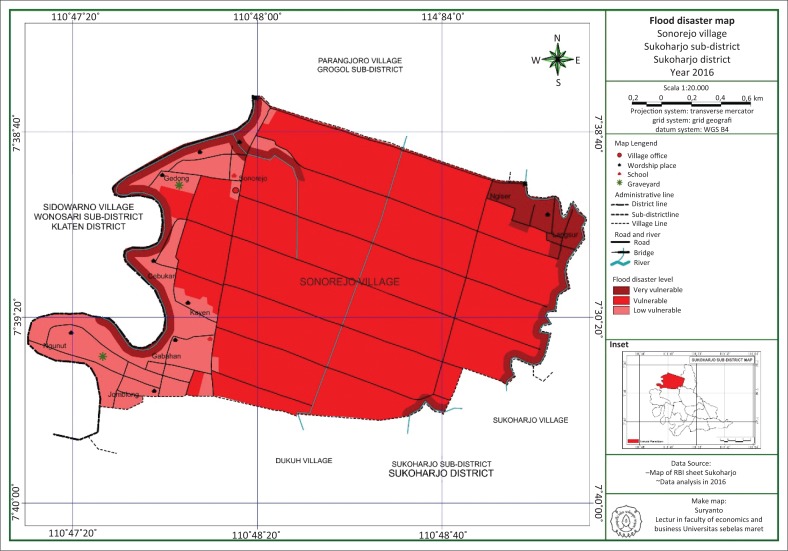
Mapping of flood-prone area in the Sonorejo Village.

Flood is a condition of overflowing of a large amount of water. Figure 1 shows the mapping of flood-prone area in the Sonorejo and Jiwo Wetan villages.

Based on Figure 1, the areas marked with dark red are the area mostly prone to flooding in the Sonorejo Village. Based on the results of the mapping using GIS with the consideration of a variable number of flood events, local curvature of the river, slope and land use, it can be seen that Langsur and Ngiser in the Sonorejo Village are two areas prone to catastrophic flooding in lowland rice fields compared with other areas.

**FIGURE 2:**
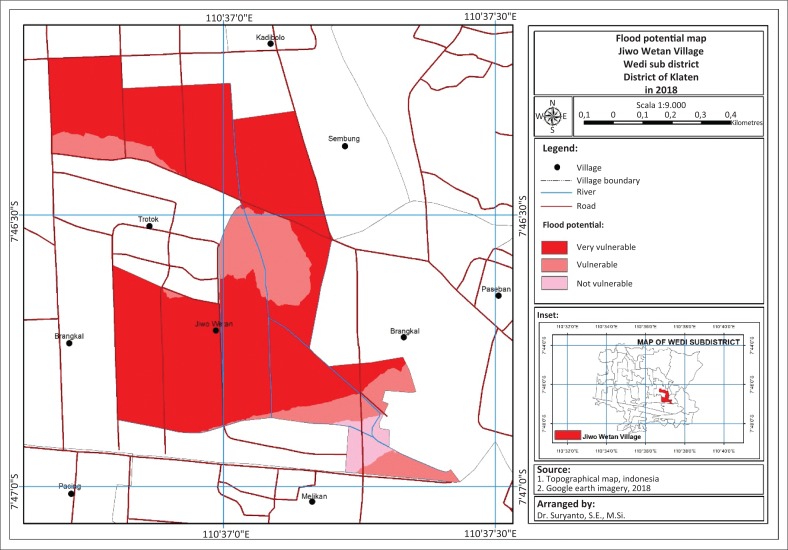
Mapping of flood-prone area in the Jiwo Wetan Village.

Based on Figure 2, the results of the mapping using GIS with the consideration of a variable number of flood events, local curvature of the river, slope and land use, it can be seen that most areas in Jiwo Wetan are marked dark red. The Jiwo Wetan Village is passed by a river which can overflow at any time if the intensity of the rain increases. Based on reports from the Local Disaster Board (Badan Penanggulangan Bencana Daerah [BPBD]), standing water can reach 1 m.

### Analysis of livelihoods vulnerability

Livelihood vulnerability index consists of seven main components, namely, SDP, LS, H, F, W, SN and ND and climate variability, and each consists of several indicators or sub-components. Each sub-component is measured by different scales that should be standardised to convert it into an index and combine it as a whole with the composite index.

The result of the standardisation of each sub-component, which is obtained by a survey of 61 families in the Sonorejo Village and 72 respondents in the Jiwo Wetan Village, is presented in Table 4.

**TABLE 4:** Livelihood vulnerability index results for the Sonorejo and Jiwo Wetan villages.

Sub-components	Composite index		Main component	Major component index	
	Sonorejo	Jiwo Wetan		Sonorejo	Jiwo Wetan
Dependent ratio	0.152	0.145	Socio-demographic profile	0.235	0.336
Percentage of female-headed households	0.115	0.368	-	-	-
Average age of female head of household	0.432	0.564	-	-	-
Percentage of household head has not attended school	0.262	0.245	-	-	-
Percentage of households with members seeking dependent care	0.213	0.356	-	-	-
Percentage of households with family member working in different community	0.295	0.456	Livelihood strategy	0.392	0.449
Percentage of households where the main source of income depends on agriculture	0.607	0.645	-	-	-
Average Agricultural Livelihood Diversification Index (range: 0.20–1)	0.274	0.245	-	-	-
Average time to health facility (min)	0.297	0.245	Health	0.263	0.245
Percentage of households’ family members with chronic illness	0.230	0.245	-	-	-
Percentage of households dependent on family farm for food	0.443	0.445	Food	0.345	0.345
Average number of months that households struggle to find food (range: 0–12)	0.036	0.045	-	-	-
Percentage of households without crops savings	0.557	0.545	-	-	-
Percentage of households using source of natural water	0.787	0.343	Water	0.274	0.145
Average time to source of water (min)	0.063	0.045	-	-	-
Percentage of households without a consistent water supply	0.082	0.045	-	-	-
The inverse of average number of litres of water stored per household (range: >0–1)	0.163	0.145	-	-	-
Average received: Given ratio (range: 0–15)	0.217	0.245	Social network	0.482	0.203
Average borrowed: Lend money ratio (range: 0.5–2)	0.574	0.234	-	-	-
Percentage of households that do not seek for assistance from their local government in the past 12 months	0.656	0.134	-	-	-
Average number of floods, in the past 5 years (0–7)	0.177	0.145	Natural disasters and climate variability	0.495	0.208
Percentage of households that did not receive warming about pending natural disaster	0.852	0.445	-	-	-
Percentage of households with injury or death as a result of the severe natural disaster in the past 5 years	0	0.033	-	-	-
Standard deviation mean average of daily maximum temperature by month	0.658	0.645	-	-	-
Standard deviation mean average of daily minimum temperature per month	0.705	0.745	-	-	-
Standard deviation mean average of precipitation by month	0.576	0.545	-	-	-

**Overall LVI**	**-**	**-**	**-**	**0.355**	**0.275**

LVI, livelihood vulnerability index.

Based on the level of vulnerability classification conducted by Hahn et al. (2009), it is known that the scale of the LVI value is set between 0 and 0.2 (not vulnerable), 0.21 and 0.40 (vulnerable or moderate) and 0.41 and 0.5 (very vulnerable). The results of the calculations in Table 4 can be interpreted as measured variables including the following.

#### Socio-demographic profile

The socio-demographic index of the Sonorejo Village is 0.235 and the Jiwo Wetan Village is 0.145. The proportion of the population of non-productive age, education pursued by the head of the family and the limitations of family members are still at a moderate level. The percentation of women as family heads is 0.115, while in the Jiwo Wetan Village it is higher at 0.368. This index shows that families led by a woman will be more vulnerable than households led by a man.

#### Livelihood strategy

The livelihood strategy index from the Sonorejo Village is 0.392, while in the Jiwo Wetan Village it is 0.499. The index of household sub-components that are based on the agricultural sector in Sonorejo sub-district is 0.607, while in the Jiwo Wetan Village it is 0.645. This shows that family dependence on the agricultural sector is more susceptible to climate change than families who do not only rely on the agricultural sector. Besides farming they also work outside the city and other economic sectors.

#### Health

The comparison of the health index numbers shown by the Sonorejo Village is 0.263, while in the Jiwo Wetan Village it is 0.245. In the health sub-component, the distance between health facilities and the number of families who have members with chronic diseases shows a moderate number. Based on the health degree vulnerability index, it can be concluded that the residents in the Sonorejo and Jiwo Wetan villages are not very vulnerable to climate change. Geographically, the Sonorejo and Jiwo Wetan villages are not isolated from health service centres.

#### Food

Based on the survey results, several families in the Sonorejo and Jiwo Wetan villages have the same characteristics in terms of food components. If the level of vulnerability in the Sonorejo Village is 0.426, then in Jiwo Wetan Village it is not significantly different at 0.445. This is because of changes in rainfall patterns and high rainfall as a result of climate change that causes flooding or inundation in the paddy fields of Sonorejo and Jiwo Wetan villages, resulting in crop failure and a decrease in rice productivity. As a result, farm households that get the main food source from their own crops become vulnerable to climate change. Based on the food sub-component index, households whose main source of food comes from their own crops do not store it for use when unpredictable conditions are the most vulnerable to climate change.

#### Water

Households in the Sonorejo Village in the water component showed a relatively moderate level of vulnerability with an index number of 0.274. As many as 78.7% of families in the Sonorejo Village use natural water sources to meet their daily water needs with consistent water availability (always available), while 8.19% or five families are recorded as not having a consistent water source. This is because their water sources sometimes have a salty or cloudy taste, so they have to find other water sources to meet their family’s water needs. The level of vulnerability in water resources in the Sonorejo Village is higher than in the Jiwo Wetan Village. Water problems in the Jiwo Wetan Village will appear during the dry season, but not significantly.

#### Social networks

The Social Network Indicator in the Sonorejo Village shows that most of the people in the Sonorejo Village did not request assistance from the local or local government during the past year (as many as 65.57% or 40 households). While in the Jiwo Wetan Village, the majority of the population also did not ask for government assistance except because of the agricultural insurance premium subsidy programme. Based on the results of the survey conducted, in Sonorejo Village the agricultural insurance programmes are still at the socialisation stage, while in the village area Jiwo Wetan has implemented the programme. The agricultural insurance programme in the Jiwo Wetan Village has given claims to farmers who have experienced crop failure.

#### Natural disasters and climate variability

The index number of ND and climate variability in the Sonorejo Village is 0.495; this figure shows that farmers in Sonorejo sub-district are very vulnerable to floods because of their location adjacent to Langsur River and lower terrain than other regions in Sonorejo sub-district. Based on the average number of floods reported in the past 6 years, as many as 85% of families did not get a warning or notification that there would be heavy rain or flooding. However, for the Jiwo Wetan region 0.445 is lower than in Sonorejo. This information is important considering the population of Jiwo Wetan when the rainy season almost always suffers from flooding because it is topographically bordered by the Dengkeng River and Pegunungan Seribu.

### Livelihood Vulnerability Index-Intergovernmental Panel on Climate Change approach

Livelihood vulnerability index/LVI-IPCC is a measure of vulnerability of farmer households in disaster-prone areas with three measurement indicators, namely, exposure, sensitivity and adaptive capacity. The formula LVI calculation formula based on the IPCC is:

$$\text{LVI}-{\text{IPCC}}_{d}=({\text{E}}_{d}-{\text{A}}_{d})*\text{Sd}.$$ [Eqn 7]

where, LVI-IPCC_*d*_ is a formula to measure the level of vulnerability of a community or community using the IPCC framework; *E*_*d*_ is a score calculation from community or community exposure; *A*_*d*_ is the calculation of the score from the capacity of a community or community and *S*_*d*_ is the calculation of the sensitivity of a community or community.

Livelihood Vulnerability Index-Intergovernmental Panel on Climate Change is an alternative method for making the alleged vulnerabilities of communities relative to the effect of climate change developed from LVI. The Final IPCC weighted LVI scores range between (−1) and (−0.4) is not vulnerable, (−0.41) and (0.3) is vulnerable or moderate and (0.31) and (1) is the most vulnerable (Table 5).

**TABLE 5:** Calculation of Livelihood Vulnerability Index-Intergovernmental Panel on Climate Change contributing factors in the Sonorejo Village and Jiwo Wetan Village.

IPCC contributing factors to vulnerability	Major components index		Major components scores	
	Sonorejo	Jiwo Wetan	Sonorejo	Jiwo Wetan
Exposure	**0.495**	**0.208**	**0.495**	**0.208**
Adaptive capacity	**3.797**	**3.762**	**0.345**	**0.342**
Socio-demographic	0.235	0.336	-	-
Livelihood vulnerability	0.392	0.449	-	-
Social network	0.482	0.245	-	-
Sensitivity	**2.657**	**2.105**	**0.295**	**0.234**
Health	0.263	0.245	-	-
Food	0.345	0.345	-	-
Water	0.274	0.145	-	-
Final IPCC weighted LVI scores	-	**-**	**0.044**	**−0.032**

Note: Bold values indicate the results of IPPC factors that contribute to vulnerability.

IPCC, Intergovernmental Panel on Climate Change; LVI, livelihood vulnerability index.

*Source*: Adapted from IPCC, 2001, *Climate change 2001: Impacts, adaptation, and vulnerability, contribution of working group II to the third assessment report*, Cambridge University Press, Cambridge

The overall value of LVI-IPCC indicated that the vulnerability of farmers’ livelihoods to flood in the Sonorejo Village goes in the category of vulnerable or moderate by 0.044 on index figure. Although Jiwo Wetan has a lower LVI than Sonorejo, LVI Jiwo Wetan is still classified as vulnerable. LVI Score in Jiwo Wetan is −0.032. The agricultural insurance programme is proven to reduce losses for farmers in Jiwo Wetan. If the social network score in Sonorejo reaches 0.482, then in Jiwo Wetan it is at the level of 0.245.

## Conclusion

According to the mapping result using GIS, rice field in the Sonorejo Village, especially in Langsur and Ngiser, has high level of vulnerability to flood because of its lower altitude than other areas.

The results of the research show that farmers in the Sonorejo Village is vulnerable to climate change by 0.363 and 0.044 index calculated by using the LVI and LVI-IPCC approach, respectively. In Jiwo Wetan, LVI is lower than in Sonorejo Village (0.275) and LVI-IPCC is also lower than in Sonorejo Village (−0.032).

The LVI index measurement results show that two regions that have the same characteristics also have different levels of vulnerability. Strengthening population capacity can be done by expanding the agricultural insurance network. Although agricultural insurance still depends on government subsidies, this policy eases the burden on farmers who experience crop failure.
